# Antihypertensive Effects of Curcumin/Turmeric Supplementation in Prediabetes and Diabetes: A Systematic Review and Meta‐Analysis of Randomised Controlled Trials

**DOI:** 10.1002/edm2.70145

**Published:** 2025-12-12

**Authors:** Hossein Bahari, Maryam Sharifi, Zahra Nejad Shahrokh Abadi, Mostafa Shahraki Jazinaki, Haniyeh Golafrouz, Zahra Asadi

**Affiliations:** ^1^ Department of Nutrition, Faculty of Medicine Mashhad University of Medical Sciences Mashhad Iran; ^2^ Department of Nutritional Sciences, School of Nutritional Sciences and Food Technology Kermanshah University of Medical Sciences Kermanshah Iran; ^3^ The Countess of Chester NHS Foundation Trust Chester UK; ^4^ Student Research Committee Mashhad University of Medical Sciences Mashhad Iran; ^5^ Rajaei Cardiovascular Medical and Research Center Iran University of Medical Sciences Tehran Iran; ^6^ Student Research Committee Shiraz University of Medical Sciences Shiraz Iran

**Keywords:** blood pressure, curcumin, diabetes, hypertension, prediabetes, turmeric

## Abstract

**Introduction:**

Hypertension is a major cardiovascular risk factor in individuals with prediabetes and type 2 diabetes (T2D). Curcumin, with its anti‐inflammatory and antioxidant properties, has emerged as a potential adjunct therapy, but its effect on blood pressure in this population remains unclear.

**Aims:**

This meta‐analysis aimed to evaluate the effects of curcumin or turmeric supplementation on systolic (SBP) and diastolic (DBP) blood pressure in adults with prediabetes or T2D.

**Methods:**

A systematic search of PubMed, Scopus, and Web of Science was conducted until August 2025. Randomised controlled trials (RCTs) investigating curcumin/turmeric supplementation on blood pressure in adults with prediabetes or T2D were included. A meta‐analysis was performed using a random‐effects model.

**Results:**

Fifteen RCTs comprising 16 treatment arms (*n* = 855 participants) were included. Pooled results indicated that curcumin/turmeric supplementation significantly reduced SBP (WMD: −2.69 mmHg; 95% CI: −3.84 to −1.55; *p* < 0.001; *I* ^2^ = 30.1%) compared to control groups. A more substantial reduction in SBP (−3.41 mmHg) was observed in the subgroup of participants with baseline hypertension (SBP ≥ 130 mmHg). However, no significant effect was found on DBP (WMD: −1.20 mmHg; 95% CI: −2.84 to 0.44; *p* = 0.15; *I* ^2^ = 84.3%). Subgroup analyses showed significant reductions in SBP in individuals with T2D or prediabetes, in those who were overweight, and with interventions using nano‐curcumin, turmeric, or curcumin with piperine at doses > 1 g/day. In addition, subgroup analysis showed that curcumin/turmeric supplementation led to a significant reduction in DBP in individuals with T2D.

**Conclusions:**

Curcumin/turmeric supplementation demonstrates a modest, yet significant reduction in SBP in individuals with prediabetes and diabetes, with a more pronounced effect in those with baseline hypertension. Further high‐quality RCTs are needed to confirm these findings and establish optimal dosing.

## Graphical abstract

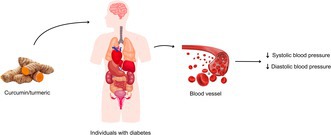

**Abbreviations**
ACC: American College of Cardiology
ACE: angiotensin‐converting enzyme
AGEs: advanced glycation end‐products
AHA: American Heart Association
AMPK: AMP‐activated protein kinase
ASCVD: atherosclerotic cardiovascular disease
BMI: body mass index
CI: confidence interval
CKD: chronic kidney disease
DBP: diastolic blood pressure
DM: diabetes mellitus
eNOS: endothelial Nitric Oxide Synthase
FPG: fasting plasma glucose
GRADE: grading of recommendations assessment, development and evaluation
HbA1c: haemoglobin A1c
HOMA‐IR: homeostatic model assessment of insulin resistance
IQR: interquartile range
MeSH: medical subject headings
NF‐κB: Nuclear Factor Kappa‐Light‐Chain‐Enhancer of Activated B Cells
NIDDM: non‐insulin dependent diabetes mellitus
NO: nitric oxide
PRISMA: preferred reporting items for systematic reviews and meta‐analyses
RAS: renin‐angiotensin system
RCT: randomised controlled trial
RoB 2: revised cochrane risk of bias tool for randomised trials
ROS: reactive oxygen species
SBP: systolic blood pressure
SD: standard deviation
SE: standard error
T2D: type 2 diabetes
TNF‐α: tumour necrosis factor‐alpha
WHO: world health organization
WMD: weighted mean difference

## 1 Introduction

Diabetes mellitus (DM) is a prevalent, multifactorial chronic disease characterised by metabolic dysregulation and has a considerable global impact. According to the World Health Organization (WHO), in 2022, nearly 450 million adults aged 30 and older, approximately 59% of all adults with diabetes, remained untreated, representing a 3.5‐fold increase in untreated cases since 1990 [1]. Annually, diabetes directly causes an estimated 1.5 million deaths, with mortality rates continuing to rise [2]. Alarmingly, nearly 48% of these deaths occur in individuals younger than 70 years, underscoring the premature mortality linked to the disease [3]. The WHO estimates that diabetes may rise to become the seventh leading cause of death globally by 2030 [4].

Diabetes mellitus encompasses several distinct subtypes, each characterised by unique etiologies and pathophysiological mechanisms [5]. Type 1 diabetes, characterised by autoimmune‐mediated beta‐cell destruction; Type 2 diabetes (T2D), associated with insulin resistance and relative insulin deficiency; neonatal diabetes, which presents in the first 6 months of life due to genetic mutations; gestational diabetes, defined by glucose intolerance with onset during pregnancy; as well as steroid‐induced diabetes, which arises secondary to prolonged corticosteroid therapy [6].

Diabetes mellitus is often accompanied by hypertension, a major and serious complication defined by a systolic blood pressure (SBP) ≥ 130 mmHg and/or a diastolic blood pressure (DBP) ≥ 80 mmHg [7]. This coexistence significantly elevates the risk of cardiovascular morbidity and mortality [8]. Chronic hyperglycemia and metabolic disturbances accelerate vascular damage, leading to both macrovascular complications, such as heart disease and stroke, and microvascular issues, including retinopathy and nephropathy [9]. Effective management of blood pressure, alongside glycemic control and lifestyle modifications, is essential to reduce these risks. Regular monitoring and early intervention remain critical components of comprehensive diabetes care [10].

A growing body of research is now focused on harnessing the power of natural products to address diverse medical conditions [11]. Among these natural compounds, curcumin, a polyphenolic compound derived from turmeric, the rhizomatous root of *Curcuma longa* , has attracted considerable attention. Curcumin constitutes approximately 75% of the curcuminoids extracted from turmeric, which belongs to the Zingiberaceae family and owes its characteristic vibrant orange‐yellow colour to these bioactive curcuminoids [12, 13]. These lipophilic polyphenolic substances make up between 2% and 8% of turmeric's total composition and are widely recognised for their potent antioxidant, anti‐inflammatory, cardioprotective, lipid‐regulating, antimicrobial, and anticancer effects [14, 15]. Extensive research has unequivocally demonstrated curcumin's therapeutic potential, highlighting its significant role in promoting human health and its ongoing prominence as a subject of scientific investigation [16].

Numerous studies have elucidated the therapeutic potential of curcumin in modulating various aspects of diabetes management. Curcumin has been shown to significantly reduce waist circumference, fat mass, hip circumference, body weight, blood glucose levels, and triglyceride levels in patients with T2D [17]. A systematic review of 20 randomised controlled trials (RCTs) further confirmed that turmeric/curcumin supplementation improves body weight and waist circumference in individuals with prediabetes [18]. Moreover, another meta‐analysis of four trials reported improvements in insulin resistance, HOMA‐IR, HbA1c, overall glycemic control, and reductions in triglycerides and total cholesterol among patients with T2D [19].

While previous meta‐analyses have examined the effects of curcumin on blood pressure in general populations with mixed results [20], a comprehensive and focused analysis in the high‐risk context of prediabetes and diabetes, where distinct pathophysiological mechanisms like chronic inflammation and endothelial dysfunction may modulate its effects, is lacking. Our study aims to fill this gap by conducting a systematic review and meta‐analysis to provide critical insights into the role of curcumin in blood pressure modulation in this specific population.

## 2 Methods

The present systematic review was conducted and reported in compliance with PRISMA protocols [21] and is registered in the PROSPERO database (registration number: CRD420251132598).

### 2.1 Search Strategy

We conducted a systematic literature search to identify all relevant RCTs published from inception through August 2025. The electronic databases searched included PubMed, Scopus, and Web of Science. The search strategy utilised a combination of Medical Subject Headings (MeSH) terms and keywords related to the population (e.g., ‘prediabetes’, ‘diabetes mellitus’, ‘type 2 diabetes’), and intervention (e.g., ‘curcumin’, ‘turmeric’, ‘curcuminoids’, ‘ *Curcuma longa* ’). The search strategy is provided as a supplement (Table S1). Additionally, the reference lists of similar review articles were manually searched and screened, and an additional search was performed using the Google Scholar search engine.

### 2.2 Eligibility Criteria

Studies were selected based on the following PICOS criteria:

- Population: Adult human participants (aged ≥ 18 years) diagnosed with prediabetes or T2D.
- Intervention: Oral supplementation with curcumin, curcuminoids, turmeric, or any bio‐enhanced form of curcumin (e.g., nano‐curcumin, phytosomal curcumin, curcumin‐piperine) in any dosage and for any duration.
- Comparison: Placebo or conventional therapy.
- Outcomes: The primary outcomes were changes in SBP and DBP.
- Study Design: RCTs of parallel or crossover design.

Exclusion criteria were: (1) non‐randomised studies, reviews, editorials, and conference abstracts; (2) studies conducted on animals or in vitro; (3) studies where the intervention was administered intravenously or topically; (4) studies that involved co‐supplementation with other active ingredients where the effect of curcumin could not be isolated; and (5) studies that were otherwise eligible but did not report baseline and post‐intervention BP values as mean ± SD for both the intervention and control groups were excluded from the quantitative synthesis.

### 2.3 Study Selection and Data Extraction

All identified records from the database searches were imported into EndNote X20 software (Clarivate Analytics) for duplicate removal. Two independent reviewers (H.G. and Z.A.) screened the remaining titles and abstracts to identify potentially eligible studies. The full texts of these articles were then retrieved and assessed in detail against the eligibility criteria. Any disagreements between the reviewers were resolved through discussion or by consultation with a third reviewer (H.B.).

Two independent researchers (Z.A. and H.G.) were collected the following data from each included study using a pre‐designed extraction form: first author's name, year of publication, country of origin, study design, sample size and number of included individuals in each group, participant characteristics (mean age, mean body mass index (BMI), and health status), features of what was received in both intervention and control groups (form, dosage, and duration), and changes in outcome levels (or baseline and post‐intervention means and SDs for SBP and DBP). Any disagreements were resolved through consultation with the third author (H.B.).

### 2.4 Risk of Bias Assessment

Two reviewers (Z.A. and H.G.) independently assessed the risk of bias for each included RCT using the revised Cochrane Risk of Bias tool for randomised trials (RoB 2) [22]. This tool evaluates bias across five domains: (1) bias arising from the randomization process, (2) bias due to deviations from intended interventions, (3) bias due to missing outcome data, (4) bias in measurement of the outcome, and (5) bias in selection of the reported result. Each domain was judged as ‘low risk,’ ‘some concerns,’ or ‘high risk.’ An overall risk of bias judgement for each study was determined based on the assessments across all domains.

### 2.5 Statistical Analysis

The meta‐analysis was performed using STATA software version 17 (Stata Corp, College Station, TX, USA). A *p*‐value of < 0.05 was considered statistically significant for all analyses. The mean change from baseline and its SD for each group were used to calculate the weighted mean difference (WMD) with a 95% confidence interval (CI) for SBP or DBP. When these values were not directly reported, they were calculated using the following formula: Mean change = (final value—baseline value); SDchange = √[(SDbaseline)^2^ + (SDfinal)^2^−(2 × *R* × SDbaseline × SDfinal)] [23]. If data were presented as 95% CIs, standard errors (SEs), or interquartile ranges (IQRs), they were converted to SDs using established methods [24]. Heterogeneity among the included studies was assessed using Cochran's Q test and the *I* ^2^ statistic. An *I* ^2^ value greater than 50% was considered to represent substantial heterogeneity. A random‐effects model was applied to pool the effect sizes due to anticipated clinical and methodological heterogeneity.

To explore heterogeneity sources, we conducted pre‐specified subgroup analyses based on: health status (prediabetes vs. T2D), geographical region (Iran vs. non‐Iran), intervention form, dosage (≤ 1 g/day vs. > 1 g/day), duration (< 12 weeks vs. ≥ 12 weeks), baseline BMI (normal, overweight, obese), baseline SBP (< 130 vs. ≥ 130 mmHg), and baseline DBP (< 80 vs. ≥ 80 mmHg). We conducted a sensitivity analysis by iteratively removing individual studies to assess their influence on the pooled effect size. Meta‐regression analysis was conducted to examine the potential linear associations between the WMDs of blood pressure and continuous variables (dosage and duration of intervention). Furthermore, a fractional polynomial model was used to explore potential non‐linear dose–response relationships. Publication bias was assessed visually using funnel plots and formally using Egger's regression test. If significant publication bias was detected, the Duval and Tweedie nonparametric ‘trim and fill’ method was applied to adjust the effect size.

### 2.6 Certainty of Evidence

We evaluated the overall certainty of evidence for each outcome using the Grading of Recommendations Assessment, Development and Evaluation (GRADE) approach [25]. Under this framework, evidence from RCTs begins as high certainty but can be downgraded to moderate, low, or very low, due to risk of bias, inconsistency, indirectness, imprecision, or publication bias.

## 3 Results

### 3.1 Study Selection

**FIGURE 1:**
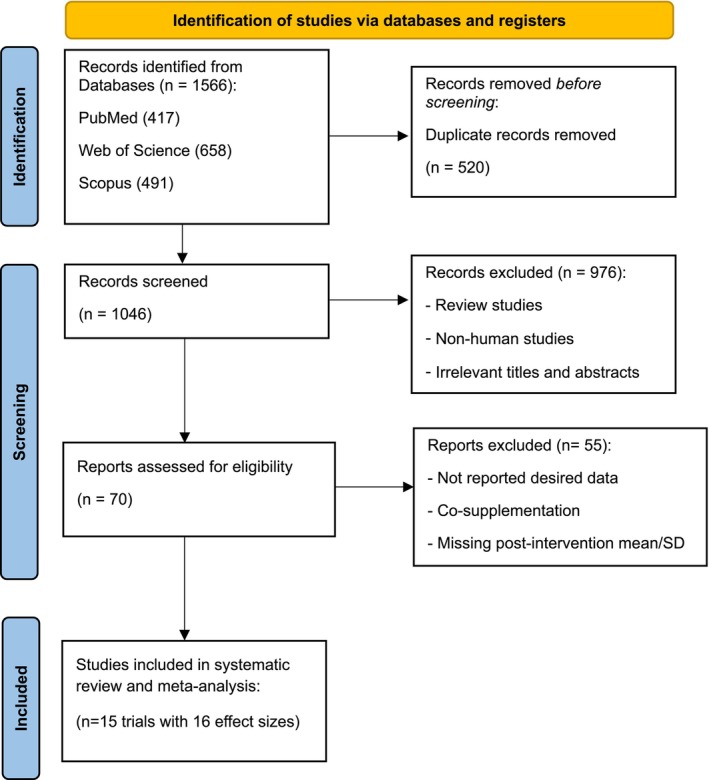
PRISMA flow chart of study selection process in the systematic review.

Out of 1566 records that were found by the initial search in databases, 520 duplicated cases were removed. The remaining 1046 papers were screened according to their titles and abstracts, resulting in the exclusion of 976 irrelevant records. The full text of 70 studies was read, which led to the exclusion of 55 papers due to not reporting required data (*n* = 51) and co‐supplementation (*n* = 4). Finally, 15 RCTs with 16 treatment arms were eligible for inclusion in this systematic review and meta‐analysis [26, 27, 28, 29, 30, 31, 32, 33, 34, 35, 36, 37, 38, 39, 40]. The study selection process is exhibited in Figure 1.

### 3.2 Study Characteristics

Eligible RCTs were conducted between 2011 [26] and 2025 [37, 38, 39, 40]. The countries of origin included Iran [26, 29, 32, 33, 35, 36, 39, 40], Mexico [27], Japan [28], India [30], USA [38], Italy [31], Brazil [34], and Egypt [37]. All of the included trials had a parallel design. Also, among the eligible studies, one was a single‐blind study [35], one had no blinding [37], one was triple‐blinded [40], while the rest were double‐blind studies. Fourteen trials included both male and female participants, while one trial [35] enrolled females only. Out of 15 eligible trials, three were conducted on individuals with prediabetes [31, 38, 40], while the others were performed on T2D patients. The interventions in eligible trials included turmeric [26, 30, 35], curcumin [27, 28, 29, 37, 38], nano‐curcumin [33, 39], phytosomal curcumin [31], curcuminoid [32], and curcumin‐piperine [34, 36, 40]. Except for one trial that applied conventional therapy in its control group [37], the rest of the included RCTs used a placebo. Furthermore, the duration of interventions in eligible studies was between 8 [26, 27, 31, 35] and 24 weeks [28]. The sample sizes of treatment arms varied between 21 [35] and 114 participants [30]. The mean age and BMI of participants in included treatment arms ranged from 42.5 [35] to 69.5 years old [28], and 24.9 [28] to 35.6 kg/m^2^ [37]. The characteristics of eligible studies are summarised in Table 1.

**TABLE 1:** Characteristics of included studies in meta‐analysis.

Studies	Country	Study design	Participant	Sex	Sample size		Trial duration (week)	Means Age		Means BMI		Intervention		
					IG	CG		IG	CG	IG	CG	Type	Dose (mg/day)	Control group
Khajehdehi et al. (2011)	Iran	R, DB, PC, Parallel	Overt type 2 diabetic nephropathy	B	20	20	8	52.9	52.6	NR	NR	Turmeric	1500	Placebo (Starch)
Jiménez‐Osorio et al. (2016)	Mexico	R, DB, PC, Parallel	Diabetic proteinuric CKD	B	28	23	8	55	56.2	29.7	27.9	Curcumin	320	Placebo (Starch)
Funamoto et al. (2019)	Japan	R, DB, PC, Parallel	Patients with IGT and NIDDM	B	15	18	24	70	69	24.90	25	Curcumin	180	Placebo
Hodaei et al. (2019)	Iran	R, DB, PC, Parallel	T2D	B	21	23	10	58	60	29.2	28.2	Curcumin	1500	Placebo (Cooked rice flour)
Srinivasan et al. (2019)	India	R, DB, PC, Parallel	T2D	B	60	54	12	51.32	49.94	27.69	26.59	Turmeric	1200	Placebo (Starch)
Shafabakhsh et al. (2020)	Iran	R, DB, PC, Parallel	Patients with diabetes on HD	B	26	27	12	58.3	56.2	27.9	27.1	Nano‐curcumin	80	Placebo
Cicero et al. (2020)	Italy	R, DB, PC, Parallel	Overweight subjects with suboptimal FPG	B	40	40	8	54	53	27.1	26.9	Phytosomal curcumin	1600	Placebo
Ebrahimkhani et al. (2020)	Iran	R, DB, PC, Parallel	T2D	B	16	19	12	56.19	52.16	31.80	30.4	Curcuminoid	500	Placebo (Maltodextrin)
Neta et al. (2021)	Brazil	R, DB, PC, Parallel	T2D	B	33	28	16	63.1	61.9	29.6	28.5	Curcumin‐Piperine	500	Placebo
Darmian et al. (2022)	Iran	R, SB, PC, Parallel	Hyperlipidemic T2D	F	11	10	8	44.33	44.22	29.3	29	Turmeric	2100	Placebo (Corn starch)
Darmian et al. (2022)	Iran	R, SB, PC, Parallel	Hyperlipidemic T2D	F	11	10	8	43.02	42.13	28.5	28.3	Turmeric + Aerobic training	2100	Placebo (Corn starch) + Aerobic training
Hosseini et al. (2024)	Iran	R, DB, PC, Parallel	T2D with hypertriglyceridemia	B	33	32	12	55.25	56.17	31.55	30.55	Curcumin‐Piperine	500	Placebo (Maltodextrin)
Mansour et al. (2025)	Iran	R, DB, PC, Parallel	Diabetic peripheral neuropathy	B	41	45	16	62.32	62.67	29.55	28.17	Nano‐curcumin	80	Placebo
Lamichhane et al. (2025)	USA	R, DB, PC, Parallel	Prediabetic Older Adults	B	14	9	12	65.5	67	32.36	29.52	Curcumin	80	Placebo
Ghazaee et al. (2025)	Iran	R, TB, PC, Parallel	Prediabetes	B	26	30	12	48.03	48.76	27.94	26.69	Curcumin‐Piperine	500	Placebo (microcrystalline cellulose)
El‐Rakabawy et al. (2025)	Egypt	R, Open, CO, Parallel	T2D with ASCVD	B	36	36	14	59.8	60.9	35.1	36.1	Curcumin + conventional therapy	1500	Conventional therapy

Abbreviations: ASCVD, atherosclerotic cardiovascular disease; BMI, body mass index; CG, control group; CKD, chronic kidney disease; CO, controlled; DB, double‐blinded; FPG, fasting plasma glucose; HD, haemodialysis; IG, intervention group; NIDDM, non‐insulin dependent diabetes mellitus; NR, not reported; PC, placebo‐controlled; R, randomised; SB, single‐blinded; T2D, type 2 diabetes; TB, triple‐blinded.

### 3.3 Risk of Bias Assessment

**FIGURE 2:**
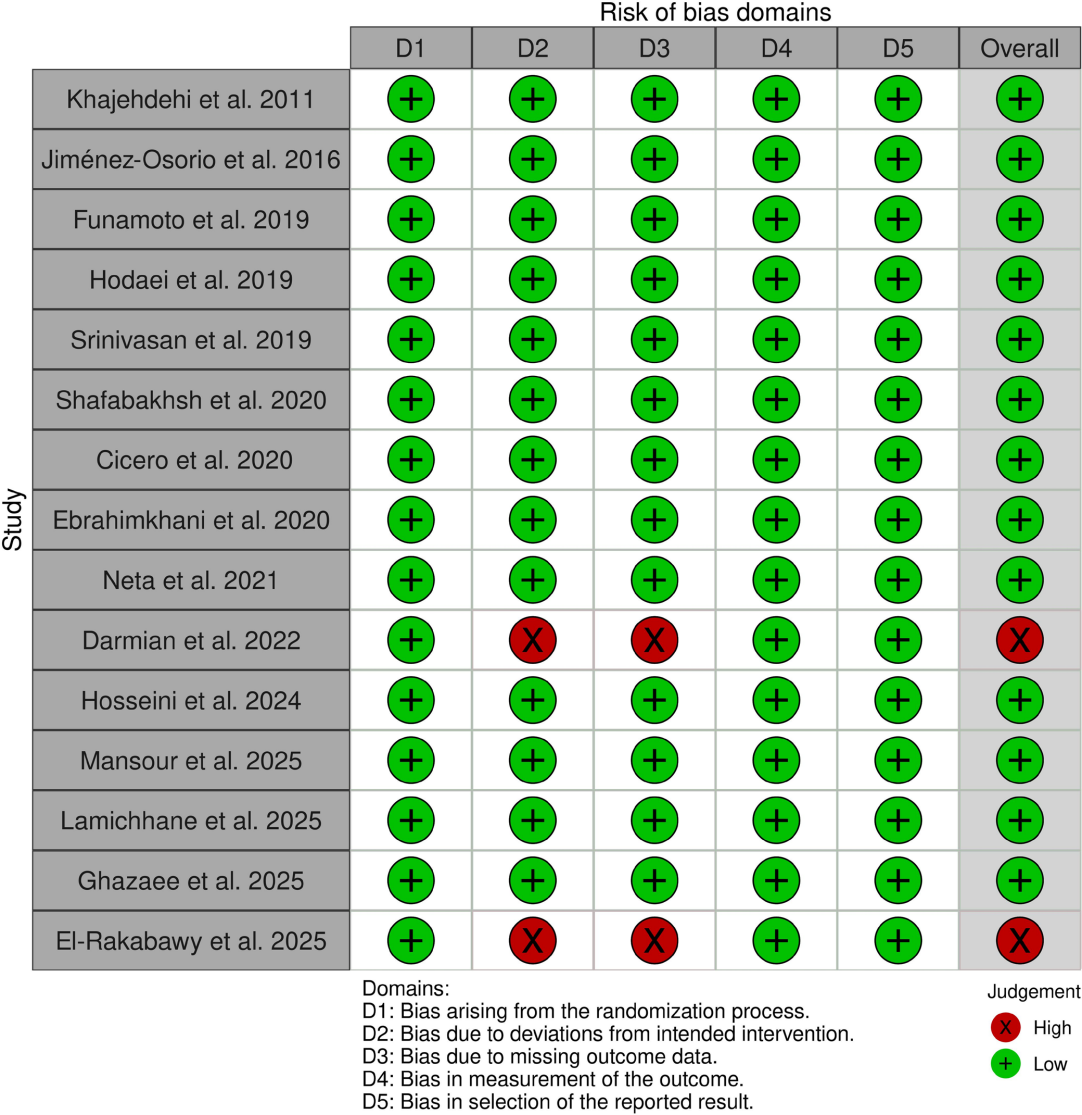
Results of risk of bias evaluation according to the Cochrane tool.

The risk of bias that was performed using the RoB 2 tool identified the general risk of bias for two included trials as high due to high risk of bias in the following two domains: bias due to deviations from intended interventions and bias due to missing outcome data [35, 37]. However, the rest of the included RCTs had low overall risk of bias. Details of risk of bias assessments in each domain are provided in Figure 2.

### 3.4 Meta‐Analysis

#### 3.4.1 Effect of Curcumin/Turmeric Supplementation on SBP

**FIGURE 3:**
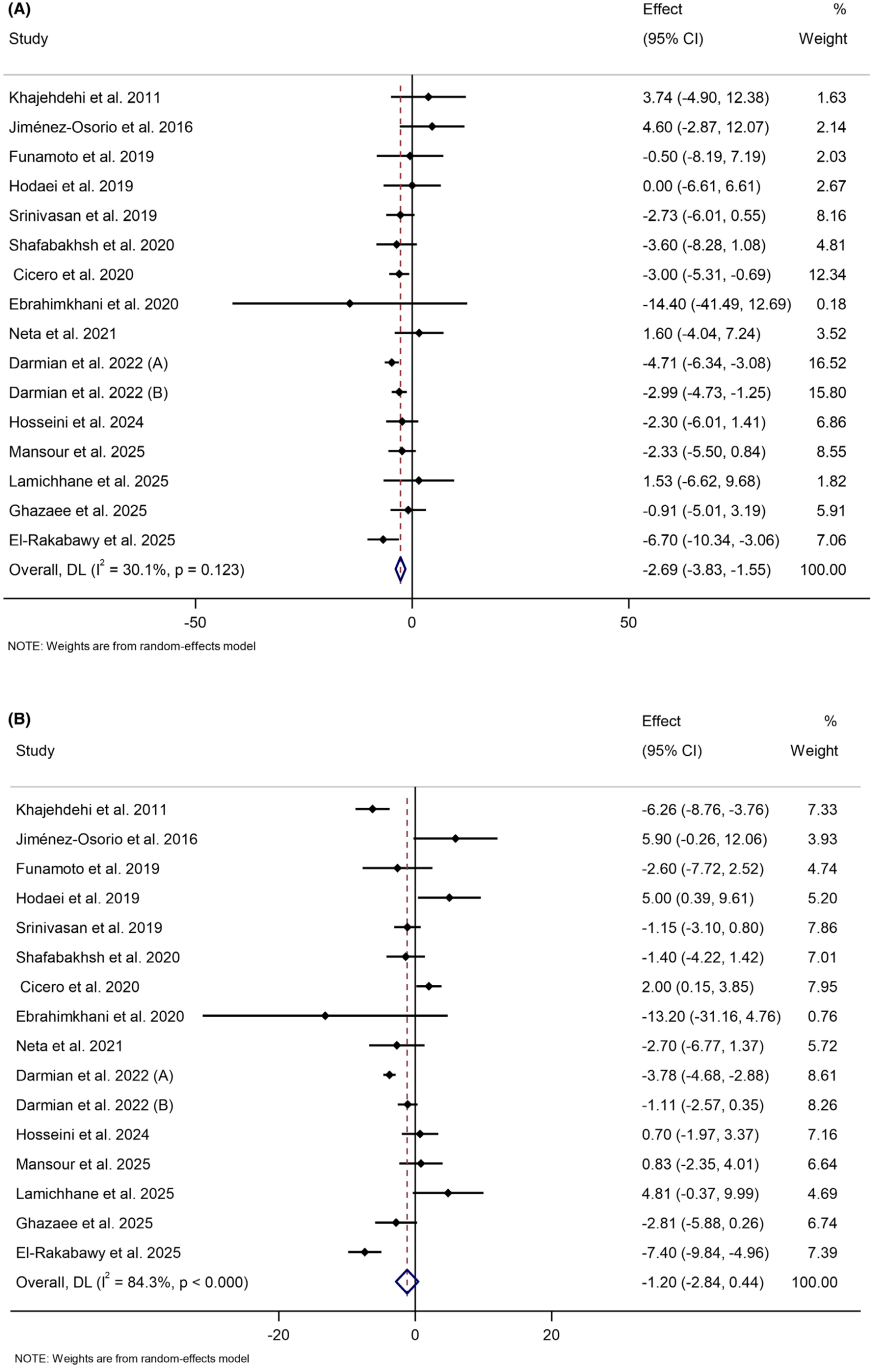
Forest plot detailing weighted mean difference and 95% confidence intervals (CIs) for the effect of curcumin/turmeric on (A) systolic blood pressure (mmHg) and (B) diastolic blood pressure (mmHg).

Pooling 16 effect sizes revealed that curcumin/turmeric supplementation led to a significant reduction in SBP compared to the control groups (WMD: −2.69 mmHg; 95% CI: −3.84 to −1.55; *p* < 0.001) (Figure 3a). Also, no significant heterogeneity was observed among the included effect sizes (*I* ^2^ = 30.1%, *p* = 0.12). Subgroup analysis, carried out to detect the source of heterogeneity, showed that curcumin/turmeric intake significantly decreased SBP in RCTs conducted in Iran, in individuals with T2D or prediabetes, in overweight individuals (25 kg/m^2^ < BMI ≤ 29.9 kg/m^2^) or those with baseline SBP ≥ 130 mmHg, as well as in trials that used nano‐curcumin, turmeric, or curcumin plus piperine, or an intervention dosage of more than 1 g/day (Table 2).

**TABLE 2:** Subgroup analyses of Curcumin/Turmeric on blood pressure in prediabetes and diabetes.

	Number of effect sizes	WMD (95% CI)	*p*	Heterogeneity		
				*p* heterogeneity	*I* ^2^	*p* between sub‐groups
Curcumin intake on SBP						
Overall effect	16	−2.69 (−3.84, −1.55)	**< 0.001**	0.123	30.1%	
Baseline SBP						
≥ 130	10	−3.41 (−4.52, −2.29)	**< 0.001**	0.24	22.1%	0.021
< 130	6	−0.48 (−2.70, 1.74)	0.673	0.597	0.0%	
Location of study						
Iran	9	−3.11 (−4.26, −1.95)	**< 0.001**	0.335	12.0%	0.408
Non‐Iran	7	−1.96 (−4.42, 0.50)	0.119	0.063	49.8%	
Trial duration (week)						
≥ 12	10	−2.57 (−4.07, −1.07)	**0.001**	0.359	9.0%	0.972
< 12	6	−2.61 (−4.49, −0.73)	**0.006**	0.055	53.9%	
Intervention dose (g/day)						
> 1	7	−3.53 (−4.88, −2.18)	**< 0.001**	0.15	36.5%	0.047
< 1	9	−1.36 (−3.03, 0.30)	0.109	0.596	0.0%	
Intervention type						
Turmeric	4	−3.38 (−5.08, −1.67)	**< 0.001**	0.152	43.2%	0.682
Curcumin	6	−1.26 (−5.72, 3.20)	0.58	0.051	54.5%	
Nano‐curcumin	2	−2.73 (−5.35, −0.11)	**0.041**	0.659	0.0%	
Curcumin plus Piperine	4	−2.09 (−3.77, −0.40)	**0.015**	0.463	0.0%	
Baseline BMI (kg/m^2^)						
Normal (18.5–24.9)	1	−0.50 (−8.18, 7.18)	0.899	—	—	0.754
Overweight (25–29.9)	10	−2.72 (−3.91, −1.54)	**< 0.001**	0.174	29.5%	
Obese (> 30)	4	−3.70 (−7.57, 0.17)	0.061	0.154	42.9%	
Health status						
Diabetes	13	−2.78 (−4.15, −1.41)	**< 0.001**	0.089	36.7%	0.672
Prediabetes	3	−2.27 (−4.22, −0.31)	**0.023**	0.441	0.0%	
Curcumin intake on DBP						
Overall effect	16	−1.20 (−2.84, 0.44)	0.152	< 0.001	84.3%	
Baseline DBP						
≥ 80	8	−0.97 (−3.44, 1.50)	0.442	< 0.001	90.0%	0.799
< 80	8	−1.41 (−3.68, 0.86)	0.224	0.001	72.0%	
Location of study						
Iran	9	−1.60 (−3.46, 0.26)	0.091	< 0.001	80.8%	0.575
Non‐Iran	7	−0.52 (−3.82, 2.79)	0.76	< 0.001	87.3%	
Trial duration (week)						
≥ 12	10	−1.66 (−3.77, 0.46)	0.125	< 0.001	74.4%	0.495
< 12	6	−0.43 (−3.26, 2.41)	0.769	< 0.001	91.6%	
Intervention dose (g/day)						
> 1	7	−2.06 (−4.43, 0.30)	0.088	< 0.001	91.4%	0.248
< 1	9	−0.27 (−2.17, 1.63)	0.782	0.044	49.6%	
Intervention type						
Turmeric	4	−2.96 (−4.95, −0.96)	**0.004**	< 0.001	84.4%	0.261
Curcumin	6	−0.12 (−6.13, 5.90)	0.97	< 0.001	88.0%	
Nano‐curcumin	2	−0.41 (−2.58, 1.76)	0.712	0.303	5.6%	
Curcumin plus Piperine	4	−0.37 (−2.82, 2.09)	0.771	0.025	67.9%	
Baseline BMI (kg/m^2^)						
Normal (18.5–24.9)	1	−2.60 (−7.72, 2.52)	0.32	—	—	0.679
Overweight (25–29.9)	10	−0.44 (−2.20, 1.32)	0.625	< 0.001	83.2%	
Obese (> 30)	4	−2.06 (−8.61, 4.49)	0.538	< 0.001	89.9%	
Health status						
Diabetes	13	−1.77 (−3.46, −0.07)	**0.041**	< 0.001	81.8%	0.196
Prediabetes	3	1.02 (−2.85, 4.89)	0.605	0.011	78.0%	

*Note:* Bold indicates statistically significant (*p*‐value < 0.05).

Abbreviations: BMI, body mass index; CI, confidence interval; DBP, diastolic blood pressure; SBP, systolic blood pressure; WMD, weighted mean differences.

#### 3.4.2 Effect of Curcumin/Turmeric Supplementation on DBP

Meta‐analysis of 16 effect sizes demonstrated no significant alteration in DBP following supplementation of curcumin/turmeric compared to control groups (WMD: −1.20 mmHg; 95% CI: −2.84 to 0.44; *p* = 0.15) (Figure 3b). However, there was significant heterogeneity among the pooled effect sizes (*I* ^2^ = 84.3%, *p* < 0.001). Subgroup analysis showed that curcumin/turmeric supplementation led to a significant reduction in DBP in individuals with T2D and in trials that used turmeric (Table 2).

### 3.5 Sensitivity Analysis

A leave‐one‐out sensitivity analysis revealed that the pooled effect sizes for the impact of curcumin/turmeric on both SBP and DBP were robust and not unduly influenced by any single study.

### 3.6 Meta‐Regression and Dose–Response Analysis

Meta‐regression showed no significant linear relationship between the feature of the intervention, including duration or dosage of the intervention, and changes in SBP or DBP levels (for all: *p* > 0.05). (Linear relations between the dosage and duration of curcumin/turmeric intake with changes in blood pressure (SBP and DBP) are presented in Figures S1 and S2, respectively). In addition, fractional polynomial modeling revealed no significant non‐linear relationship between the dosage or duration of the intervention and changes in levels of SBP or DBP (for all: *p* > 0.05) (Non‐linear relations between the dosage and duration of curcumin/turmeric intake with changes in blood pressure (SBP and DBP) are exhibited in Figures S3 and S4, respectively).

### 3.7 Publication Bias

The results of Egger's regression test, as well as visual inspection of funnel plots, indicated a significant publication bias among pooled effect sizes for investigating the influence of the curcumin/turmeric intake on SBP levels (*p*_Egger_ = 0.02) (Figure S5). Due to significant publication bias for SBP, the Trim and Fill method was performed to adjust for the influence of the potential missing studies due to publication bias. Based on the Trim and Fill analysis, four potentially missing studies were imputed. The observed pooled effect size, followed by trimming, was −2.76 mmHg (95% CI: −3.84 to −1.68), while after imputing the four missing studies, it changed to −3.27 mmHg (95% CI: −4.31 to −2.23). However, no significant publication bias was detected between the effect sizes that were pooled to assess the impact of curcumin/turmeric supplementation on DBP levels (*p*_Egger_ = 0.21).

### 3.8 GRADE Analysis

Based on the GRADE framework, the levels of evidence certainty are assessed. The levels of evidence certainty for SBP were identified as moderate due to serious publication bias. However, due to a very serious inconsistency in the evidence investigating the impacts of curcumin/turmeric supplementation on DBP, the certainty levels were downgraded to low. The details of the centenary assessment of evidence are provided in the GRADE profile (Table 3).

**TABLE 3:** GRADE profile of Curcumin/Turmeric on blood pressure in prediabetes and diabetes.

Outcomes	Risk of bias	Inconsistency	Indirectness	Imprecision	Publication bias	Quality of evidence
SBP	No serious limitation	No serious limitation	No serious limitation	No serious limitation	Serious limitation	⊕ ⊕ ⊕◯ Moderate
DBP	No serious limitation	Very serious limitation^a^	No serious limitation	No serious limitation	No serious limitation	⊕ ⊕ ◯◯ Low

^a^ There is high heterogeneity (*I* ^2^ > 75%).

## 4 Discussion

**FIGURE 4:**
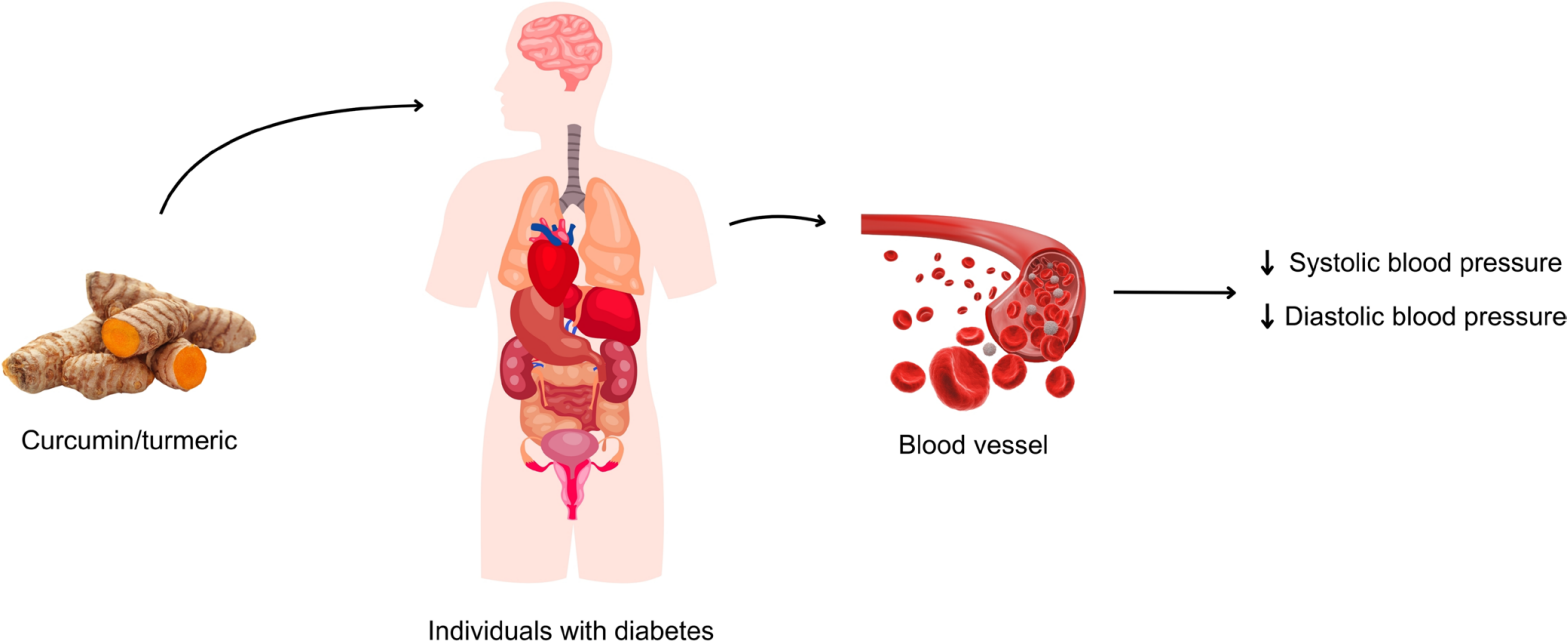
Pooled data from randomised controlled trials demonstrate that supplementation with curcumin or turmeric leads to a significant reduction in systolic blood pressure (SBP) in individuals with prediabetes and type 2 diabetes. While the overall effect on diastolic blood pressure (DBP) was not significant, a pre‐specified subgroup analysis showed a significant reduction in DBP specifically in patients with type 2 diabetes.

This meta‐analysis represents the first comprehensive effort to evaluate the impact of curcumin supplementation on blood pressure among individuals with prediabetes and type 2 diabetes. The analysis synthesises data from 15 randomised controlled trials, encompassing a total of 855 participants. The results demonstrated that curcumin supplementation led to a statistically significant, though modest, improvement in SBP (Figure 4). Subgroup analyses showed that curcumin/turmeric intake significantly decreased SBP in RCTs conducted in Iran, in individuals with T2D or prediabetes, in overweight individuals or those with baseline SBP ≥ 130 mmHg, as well as in trials that used nano‐curcumin, turmeric, or curcumin plus piperine, or an intervention dosage of more than 1 g/day. However, the significant effect observed specifically in trials conducted in Iran should be interpreted with caution, as this finding is likely confounded by the concentration of high‐dose trials in that region, rather than indicating a distinct ethnic or geographic response. According to global research, the number of people with prediabetes is rising rapidly, with projections exceeding 470 million individuals by 2030, thereby placing a greater strain on healthcare systems [41]. The prevalence of T2D is expected to increase as well, with projections indicating a rise to around 7079 cases per 100,000 people by 2030 [42]. T2D constitutes over 80% of all diabetes cases globally [43]. Therefore, enhancing prediabetes surveillance is recommended to facilitate the effective implementation of diabetes prevention policies and interventions. Lifestyle modification, such as adherence to healthy diets and regular physical activity, plays a crucial role [44].

Studies indicate that oxidative stress and inflammation are the two main factors contributing to elevated blood pressure in individuals with prediabetes and diabetes. Elevated levels of reactive oxygen species (ROS) contribute to vascular dysfunction by damaging endothelial cells, promoting inflammation, and impairing nitric oxide bioavailability [45]. Additionally, chronic inflammatory processes, marked by increased circulating pro‐inflammatory cytokines such as TNF‐α, exacerbate vascular injury and insulin resistance [46]. The interplay between oxidative stress and inflammation is a key pathogenic mechanism in hypertension and explains its common comorbidity with diabetes.

Recent meta‐analytical evidence presents inconsistent findings concerning the efficacy of curcumin supplementation on blood pressure regulation. One meta‐analysis, which synthesized data from 11 studies, reported that curcumin/turmeric supplementation had no significant impact on SBP and DBP levels. However, the prolonged administration of curcumin/turmeric (≥ 12 weeks) may result in significant reductions in SBP among adult populations, while it has no significant influence on DBP levels [20]. Similarly, another meta‐analysis that encompassed 17 RCTs confirmed the inability of curcumin supplementation to change SBP and DBP in adults, while in this review, the longer duration of (≥ 12 weeks) of curcumin supplementation led to a significant reduction in DBP levels, while SBP levels were not significantly changed [47]. Furthermore, a meta‐analysis comprising 10 studies showed curcumin significantly decreased the DBP in patients with metabolic disorders, while no significant alteration in SBP was observed [48]. The inconsistency in these findings may stem from heterogeneity in intervention duration and curcumin dosage across studies. Furthermore, while previous meta‐analyses have employed broader inclusion criteria, the present study offers a more specific analysis by focusing exclusively on RCTs within prediabetes and diabetes populations, now incorporating a larger body of evidence with 15 trials.

Several theories explain the mechanisms by which curcumin supplementation lowers SBP in these patients:

- Anti‐inflammatory and Antioxidant Effects: Curcumin exhibits strong anti‐inflammatory and antioxidant properties by reducing pro‐inflammatory cytokines (e.g., TNF‐α) and decreasing oxidative stress markers, both of which are central to vascular dysfunction and hypertension [49]. This includes the inhibition of the NF‐κB pathway, which mitigates chronic inflammation and endothelial damage [50].
- Improvement of Endothelial Function: Curcumin activates AMPK and upregulates endothelial nitric oxide synthase (eNOS) activity, enhancing nitric oxide (NO) bioavailability [51, 52]. This promotion of vasodilation consequently reduces vascular resistance.
- Enhancement of Metabolic Health: By decreasing insulin resistance and elevating adiponectin levels, curcumin improves insulin sensitivity, thereby addressing key metabolic drivers of elevated blood pressure [53].
- Inhibition of Pathogenic Pathways: Curcumin counters the harmful effects of advanced glycation end‐products (AGEs), which cause vascular stiffness and hypertension in diabetes, by inhibiting their formation and protecting vessel elasticity [54].
- Modulation of the Renin‐Angiotensin System (RAS): Curcumin modulates the RAS by suppressing angiotensin‐converting enzyme (ACE) activity, thereby lowering the production of angiotensin II, a potent molecule that induces vasoconstriction and raises blood pressure [51].

Through these multifaceted actions, reducing inflammation and oxidative stress, enhancing NO production, preventing vascular damage, and regulating hormonal blood pressure control, curcumin effectively improves vascular health and lowers SBP, reducing cardiovascular risks in individuals with prediabetes and diabetes.

The combined evidence from this study suggests that curcumin supplementation may serve as a promising adjunctive strategy for improving SBP. The overall reduction of −2.69 mmHg, while modest, is of clinical interest as it is comparable to the effects of established non‐pharmacological interventions like the DASH diet [55] and approaches the lower end of efficacy for first‐line, low‐dose antihypertensive medications [56]. Although this effect may fall below the minimal clinically important difference, often defined as a 5 mmHg decrease [57], our subgroup analysis revealed a more substantial and potentially clinically relevant reduction of −3.41 mmHg specifically in participants with baseline hypertension (SBP ≥ 130 mmHg). This finding is crucial, as it identifies a target population, diabetic and prediabetic patients with hypertension, who are likely to derive the greatest benefit from this intervention. Consequently, well‐designed, long‐term clinical trials focusing on this hypertensive subgroup are essential to thoroughly validate and define the role of curcumin as an adjunctive therapy.

### 4.1 Strengths and Limitations

The present GRADE‐assessed meta‐analysis offers several notable strengths. It specifically focuses on the impact of curcumin supplementation on blood pressure in individuals with prediabetes and diabetes, which enhances the relevance and applicability of its findings to these high‐risk populations. The inclusion of 15 trials provides a more robust estimate of the effect than previously available. Additionally, the use of the rigorous GRADE framework to evaluate the quality of evidence adds robustness and transparency to the conclusions drawn. It also considers different forms of curcumin, including turmeric, standard curcumin, nano‐curcumin, and curcumin combined with piperine, providing a comprehensive evaluation of its various formulations.

Nevertheless, this review has several limitations. Substantial heterogeneity was observed across the included studies regarding intervention duration, dosage, and participant demographics (e.g., age, ethnicity, and baseline health). This heterogeneity was particularly high for the DBP outcome (*I* ^2^ > 84%), which led to a downgrade in the certainty of evidence. The variability in DBP may stem from several factors, including differing patient comorbidities (e.g., varying degrees of renal function), inconsistencies in blood pressure measurement methods, or the possibility that curcumin's primary mechanisms have a less consistent effect on diastolic pressure. The wide prediction interval for DBP underscores that the effect in a new study could vary significantly, and these results should be interpreted with caution. Moreover, the presence of publication bias for SBP, with a tendency to report studies showing positive outcomes, may have influenced the overall conclusions, though the Trim and Fill adjustment suggested a stronger effect.

To address these limitations, future research should prioritize conducting well‐designed, large‐scale, multicenter RCTs with diverse populations and longer follow‐up periods. Additionally, efforts should be made to include unpublished or negative results to minimize bias, and standardized protocols should be established to ensure consistency in curcumin formulations, dosages, and outcome measurements across studies.

## 5 Conclusion

In conclusion, this meta‐analysis demonstrates that curcumin supplementation significantly improves SBP in individuals with prediabetes and T2D, while no significant changes were detected in DBP levels. Subgroup analysis showed a significant reduction in SBP levels in individuals who are overweight, of Iranian nationality, those who received turmeric, nano‐curcumin, or curcumin plus piperine, or those who received supplementation exceeding 1 g/day. Additionally, subgroup analysis showed that curcumin supplementation led to a significant decrease in DBP levels in participants with T2D or those who consumed turmeric. While these findings position curcumin as a promising adjunctive therapy for blood pressure management in specific prediabetes and T2D subpopulations, large‐scale, well‐designed RCTs are necessary to confirm these effects and fully establish optimal dosing and treatment strategies.

## Author Contributions

**Haniyeh Golafrouz:** investigation, data curation. **Mostafa Shahraki Jazinaki:** writing – original draft, writing – review and editing. **Hossein Bahari:** conceptualization, writing – original draft, writing – review and editing, methodology, validation, formal analysis, software, project administration. **Maryam Sharifi:** Writing – original draft, Writing – review and editing, Visualization. **Zahra Asadi:** Investigation, Data curation. **Zahra Nejad Shahrokh Abadi:** Writing – review and editing.

## Funding

The authors have nothing to report.

## Ethics Statement

The authors have nothing to report.

## Consent

The authors have nothing to report.

## Conflicts of Interest

The authors declare no conflicts of interest.

## Supporting information


**Figure S1:** Random‐effects meta‐regression plots of the association between mean changes in (A) SBP (mmHg) and (B) DBP (mmHg) and curcumin/turmeric dosage (mg/day).
**Figure S2:** Random‐effects meta‐regression plots of the association between mean changes in (A) SBP (mmHg) and (B) DBP (mmHg) and intervention duration (weeks).
**Figure S3:** Dose–response relations between dosage (mg/day) of curcumin/turmeric supplementation and mean difference in SBP (mmHg) (A) and DBP (B) (mmHg).
**Figure S4:** Dose–response relations between durations (weeks) of curcumin/turmeric supplementation and mean difference in SBP (mmHg) (A) and DBP (mmHg) (B).
**Figure S5:** Funnel plots for the effect of curcumin/turmeric on (A) systolic blood pressure and (B) diastolic blood pressure.

**Table S1:** edm270145‐sup‐0002‐TableS1.docx.

## Data Availability

The data that support the findings of this study are available from the corresponding author upon reasonable request.
