# Supplementary material for: Antihypertensive Effects of Curcumin/Turmeric Supplementation in Prediabetes and Diabetes: A Systematic Review and Meta‐Analysis of Randomised Controlled Trials
Source: Endocrinol Diabetes Metab. 2025 Dec 12;9(1):e70145. doi: 10.1002/edm2.70145 (PMC12701325; doi:10.1002/edm2.70145)
Supplement: Supplementary file 1 — Figure S1: Random‐effects meta‐regression plots of the association between mean changes in (A) SBP (mmHg) and (B) DBP (mmHg) and curcumin/turmeric dosage (mg/day). Figure S2: Random‐effects meta‐regression plots of the association between mean changes in (A) SBP (mmHg) and (B) DBP (mmHg) and intervention duration (weeks). Figure S3: Dose–response relations between dosage (mg/day) of curcumin/turmeric supplementation and mean difference in SBP (mmHg) (A) and DBP (B) (mmHg). Figure S4: Dose–response relations between durations (weeks) of curcumin/turmeric supplementation and mean difference in SBP (mmHg) (A) and DBP (mmHg) (B). Figure S5: Funnel plots for the effect of curcumin/turmeric on (A) systolic blood pressure and (B) diastolic blood pressure. [file EDM2-9-e70145-s002.docx]

**A)**

**B)**

**Supplementary Figure 1.** Random-effects meta-regression plots of the association between mean changes in A) SBP (mmHg) and B) DBP (mmHg) and curcumin/turmeric dosage (mg/day).

**A)**

**B)**

**Supplementary Figure 2.** Random-effects meta-regression plots of the association between mean changes in A) SBP (mmHg) and B) DBP (mmHg) and intervention duration (weeks).

**A)**

**B)**

**Supplementary Figure 3.** Dose-response relations between dosage (mg/day) of curcumin/turmeric supplementation and mean difference in SBP (mmHg) (A) and DBP (B) (mmHg).

**A)**

**B)**

**Supplementary Figure 4.** Dose-response relations between durations (weeks) of curcumin/turmeric supplementation and mean difference in SBP (mmHg) (A) and DBP (mmHg) (B).

**A)**

**B)**

**Supplementary Figure 5.** Funnel plots for the effect of curcumin/turmeric on A) systolic blood pressure and B) diastolic blood pressure.
