# Supplementary material for: Antihypertensive Effects of Curcumin/Turmeric Supplementation in Prediabetes and Diabetes: A Systematic Review and Meta‐Analysis of Randomised Controlled Trials
Source: Endocrinol Diabetes Metab. 2025 Dec 12;9(1):e70145. doi: 10.1002/edm2.70145 (PMC12701325; doi:10.1002/edm2.70145)
Supplement: Supplementary file 2 — Table S1: edm270145‐sup‐0002‐TableS1.docx. [file EDM2-9-e70145-s001.docx]

**Supplementary Table 1. Search strategy**

| **Search strategy** | **Database** |
| --- | --- |
| ((((((("curcumin"[Title/Abstract]) OR ("turmeric"[Title/Abstract])) OR (Mervia[Title/Abstract])) OR ("diferuloylmethane"[Title/Abstract])) OR ("curcuma longa"[Title/Abstract])) OR ("nanocurcumin"[Title/Abstract])) AND ((((((("diabetes"[All Fields]) OR ("prediabetes"[All Fields])) OR ("diabetic"[All Fields])) OR ("diabetics"[All Fields])) OR ("prediabetic"[All Fields])) OR ("prediabetics"[All Fields])) OR ("diabesity"[All Fields]))) AND ("Intervention" OR "RCT" OR "controlled trial" OR randomised OR randomized OR random OR randomly OR placebo OR "clinical trial" OR blinded OR trials OR "Cross-Over" OR parallel) | PubMed  (n=417) |
| ( TITLE-ABS-KEY ( curcumin OR turmeric OR Mervia OR diferuloylmethane OR curcuma longa OR nanocurcumin ) AND TITLE-ABS-KEY ( diabetes OR prediabetes OR diabetic OR diabetics OR prediabetic OR prediabetics OR diabesity ) AND ALL ( Intervention OR RCT OR controlled trial OR randomised OR randomized OR random OR randomly OR placebo OR clinical trial OR blinded OR trials OR Cross-Over OR parallel ) ) | Scopus  (n=491) |
| **1#**  ALL=(curcumin OR turmeric OR Mervia OR diferuloylmethane OR curcuma longa OR nanocurcumin)  **2#**  ALL=(diabetes OR prediabetes OR diabetic OR diabetics OR prediabetic OR prediabetics OR diabesity)  **3#**  ALL=(Intervention OR RCT OR controlled trial OR randomised OR randomized OR random OR randomly OR placebo OR clinical trial OR blinded OR trials OR Cross-Over OR parallel)  **4# = 1# AND 2# AND 3#** | Web of Science  (n=658) |
